# Smoking, smoking cessation and tobacco control in rural China: a qualitative study in Shandong Province

**DOI:** 10.1186/1471-2458-14-916

**Published:** 2014-09-04

**Authors:** Jian Wang, Chenghui Li, Chongqi Jia, Yanxun Liu, Junjie Liu, Xiaona Yan, Yufeng Fang

**Affiliations:** Key Lab for Health Economics and Policy Research, Ministry of Health, Shandong University, Jinan, Shandong 250012 P. R. China; Center for Health Management and Policy, Shandong University, Jinan, Shandong 250012 P. R. China; Division of Pharmaceutical Evaluation and Policy, University of Arkansas for Medical Sciences Collage of Pharmacy, 4301 West Markham Street, Slot #522, Little Rock, AR 72205 USA; Department of Epidemiology and Health Statistics, Shandong University, Jinan, Shandong 250012 P. R. China

**Keywords:** Smoking cessation, Rural residents, China

## Abstract

**Background:**

Smoking prevalence is high in China and even higher among rural residents. The aims of this study were: 1) to gain insights into the motivations of tobacco use and barriers to smoking cessation among rural village residents; 2) to understand the current tobacco control measures in the rural villages and barriers encountered or perceived for implementation.

**Methods:**

Qualitative semi-structured face-to-face interviews and focus group discussions were conducted of 59 rural villagers including 37 village residents, 10 village leaders and 12 village doctors in three counties in Shandong Province, China.

**Results:**

Smoking initiation was most often out of curiosity when seeing others smoke, but pressure from cigarette sharing and gifting custom was the major barrier to smoking cessation. The most important reason for quitting successfully was a detrimental health problem. Although many attempted to quit at the advice of other family members, relapses were common and few were able to quit completely and for long-term unless accompanied by significant health issues. Although doctor’s advice to quit is effective, many doctors do not offer advice to all patients. There is a lack of true understanding of the harm of smoking and second-hand smoking among the villagers and a lack of access to and knowledge of effective smoking cessation tools among both smokers and village doctors. Tobacco control activities at villages were rare and infrequent.

**Conclusions:**

This study highlighted the need to develop tobacco control measures that reflect the unique culture in rural China. Smoking cessation measures are not likely to achieve large scale effect unless the prevailing cigarette sharing and gifting custom is drastically changed. More educations of the hazards of smoking and second-hand smoking to village residents and educations of effective smoking cessation treatment to both village residents and healthcare providers are needed.

## Background

Tobacco control in China plays a critical role in global tobacco control efforts. China remains the largest tobacco producer and consumer in the world. According to the 2008 World Health Organization (WHO) report, since 1998, China has been manufacturing and consuming over 1.5 trillion cigarette sticks annually [1]. Overall, nearly one third of adults 15 years or older use some tobacco products (94.7% are manufactured cigarettes) [2]. Males dominate the tobacco users with an estimated prevalence rate of 52.9% by the latest nationally representative survey in 2010, compared to only 4% among females [3, 4]. Although these estimates represent a slight decrease from an earlier report in 2002 [5], the total number of adult users are still over 300 million in 2010 [4], accounting for nearly one third of the world’s smokers [6].

China is the world’s most populous country with 1.3 billion people and more than half (51.7%) of them live in the rural areas [7]. Smoking prevalence is higher among rural residents (29.8% vs. 26.1% among urban residents) [3], and particularly among adult males (56.1% vs. 49.2% among urban residents) [4]. Among adult males, agriculture workers had higher smoking prevalence than males working in other occupations with over 60% of them smoke [3]. While smoking prevalence among rural residents has been monitored periodically [4, 5, 8], few studies have examined the smoking and cessation behavior of the rural residents, and even fewer studies have conducted among rural villagers. Furthermore, existing studies of rural residents mostly are quantitative studies using questionnaires [6, 9–11], with a few exceptions [12, 13]. While quantitative studies using questionnaires have the advantage of being able to assess factors associated with smoking behaviors in a large sample, the factors that can be explored are limited to what have been included in the questionnaires. Qualitative studies, although with their own limitations, can offer far more in-depth insights into the attitudes, perceptions, and beliefs of the respondents.

Given the high prevalence of smoking and larger number of smokers among rural villagers, developing strategies to control smoking in this population is crucial to curb smoking epidemic and reduce smoking-related morbidity and mortality in China. However, implementing smoking cessation efforts may face unique challenges in rural areas because of the overall lower education levels, dispersed geographic areas, deeply rooted tobacco use tradition and customs, as well as limited access to medical care. While effective smoking cession strategies have been developed and tested in western countries [14, 15] or urban areas in China [16, 17], there is little evidence in the wider literature upon which to develop strategies that target specifically towards rural population in China.

In view of such gap in the knowledge, a qualitative study of rural villagers including residents, village leaders and village doctors in three counties in Shandong Province was conducted. The aims of this study were: 1) to gain insights into the motivations of tobacco use and barriers to smoking cessation among rural village residents; 2) to understand the current tobacco control measures in the rural villages and barriers encountered or perceived for implementation. With the knowledge gained from this study, it is hoped that targeted and sustainable tobacco control policies that will tailor to the needs and environment of rural villages in China may be developed.

## Methods

### Study population

This study was conducted in Shandong Province, which is located on the northeast coast of China. Subjects are recruited from three counties: Pingyin county of Jinan, Junan county of Linyi, and Liangshan county of Jining. Jinan, the capital city of Shandong Province, is located in the east central part of the province. Pingyin is about 87 km south west of Jinan with roughly 370,000 residents, two thirds of them reside in rural areas; the annual per capita fiscal revenue in 2009 was about 2,500 Chinese Yuan. Liangshan is about 275 km south west of Jinan with roughly 750,000 residents and four fifths of them are rural residents; the annual per capita fiscal revenue in 2009 was approximately 1,300 Chinese Yuan. Junan is about 312 km south east of Jinan with approximately 1 million residents and nearly 90% of them are rural residents; the annual per capita fiscal revenue in 2009 was approximately 400 Chinese Yuan. These counties are part of the Health Research Base established in 2009 through an agreement between Shandong University and Centers for Diseases Control and Prevention (CDC) of Jinan, Linyi, and Jining to promote the health of rural residents.

For this study, subjects were purposeful recruited and recruitment efforts were coordinated by relevant directors of local healthcare administration departments. Within each county, three closely located villages were selected for convenience and ease of organization and coordination. Within each of the 9 villages, a minimum of one village leader, one village doctor, and two village residents were required to ensure representation of views from all key stakeholders and from all villages. Village leaders and village doctors were recruited because they are the key informants of villagers’ behavior including their smoking habits and are also the implementers of tobacco control policy in the villages. Village leaders and village doctors were then asked to recommend village resident participants based on three criteria: 1) lived in the village for at least 3 years (so that he/she would be familiar with the local culture and smoking prevalence of their fellow villagers); 2) is a current or former smoker or suffering from second-hand smoking; 3) must be literate and can clearly communicate their thoughts. Current smokers were defined as those who have smoked at least 100 cigarettes in their lifetime and smoked at least one cigarette during the 30 days prior to recruitment. Former smokers were defined as those who have smoked at least 100 cigarettes in their lifetime but did not smoke during the 30 days prior to recruitment. Nonsmokers were those who never smoked in their life time. When possible, diversity in participants’ characteristics were maximized by recruiting residents who were of different age, gender, smoking status and education to achieve balanced representation of views, maximize information saturation, and minimize selection bias.

### Study methods

This study utilized qualitative study design. Data were collected through both face-to-face semi-structured interviews and focus group discussions (FGD). Village leaders and doctors were interviewed individually in their offices or clinics because they are the key informants and individual interviews are best to uncover the in-depth knowledge, perception and attitude of them. Village residents were initially planned to be interviewed through FGDs only. FGDs allow more efficient collection of information from multiple village residents and the interactive group discussions during FGDs can also provide valuable cross-validation of reported information by participants. However, it became obvious during field work that group dynamics in several discussion groups inhibited full participation by some discussants. Consequently, semi-structured face-to-face interviews with 10 additional rural residents were conducted to allow participants to speak freely about their experiences in a more confidential manner. We reached information saturation and redundancy [18].

On-site visits by research staff were conducted in August 2010. Research staff visited the three counties sequentially for five days each. Interviews/FGDs were coordinated by relevant directors of local health care administration departments at the convenience of the participants. The individual interviews were conducted by four field research staff (JW, JL, XY, YF) with two per interview on a rotating basis and each interview lasted about 25–60 minutes. FGDs were facilitated by two field research staff (JW and JL). Four FGDs were conducted with two FGDs in Pingyin county and one FGD each in the other two counties. In Pingyin county, we conducted one FGD of residents from one village and another FGD of residents from the other two villages. Each FGD had 6–9 discussants and lasted 45–90 minutes.

To ensure quality, prior to the interviews or FGDs, all field research staff received training on interview techniques and relevant information of this project. Topic guides were used to guide the interviews and discussions, which were developed after repeated deliberation, revised based on existing literature and experts’ advice, and pilot tested for its completeness and cohesiveness. The field research staff were required to be familiar with the topic guides to the extent that they could memorize them. During each interview or discussion, one interviewer/facilitator moderated the discussion and the other took notes. If the moderating interviewer/facilitator missed some questions, the other would follow-up on these questions. Interviews/FGDs were also audio-recorded after obtaining permissions of the interviewees/discussants. At the end of each field day, field research staff discussed and analyzed the information collected; any missing or unclear information were followed up the next day.

### Data analysis

Data collected were recorded, transcribed, and analyzed thematically using a “framework approach” [19–21] through a 5-stage process: 1) familiarizing with the raw data through reading field notes and interactive listening and transcribing of the tape recording and breaking the transcripts down into single-quote data; 2) identifying a thematic framework for coding data from the topic guides and inspections of the notes and transcripts; 3) summarizing the data units and giving a key concept; 4) organizing similar concepts into sub-themes using a matrix; 5) mapping the relationships between different major themes by interpreting the data set as a whole. Table 1 shows an example of how key concepts, subthemes, and major themes are developed. The themes were first developed independently by JW and JL through an inductive process. Then all field research staff (JW, CJ, YL, JL, XY, YF) discussed the themes and findings. Disagreement were resolved by further discussion. An independent analyst (CL) was used to further review the data and contest the themes. Additional source of triangulation was through systematically comparing findings from the individual interviews with findings from the FGDs [22]. The interpretation and the policy relevance of the findings were discussed among all authors. Per the journal's policy, our reporting adheres to the RATS guidelines for reporting qualitative studies.

**Table 1:** **Example of data analysis**

Data units	Key concept	Subtheme	Major theme
“Most of smokers who are beginners are adolescents, who started to smoke mainly out of curiosity and were attracted by the ‘cool’ appearance of smoking a cigarette.” (Village leader, Pingyin county, Current smoker)	Curiosity and attracted by the “cool” appearance	Curiosity	Why do people start to smoke?
“The reasons that I started smoking was one curiosity, two because of the many friends I had. When seeing others smoking, I could not help but smoked too.” (Village doctor, Liangshan county, current smoker)	Curiosity and imitating friends		
“I don’t enjoy the smell of cigarettes. But I found that the ‘smoking pose’ with two fingers gently gripping the cigarette, inhaling and blowing out smoke rings were very attractive and charming when watching smokers doing that on TV.” (Village resident, Liangshan county, current smoker)	Attracted by the “cool” appearance		
“I started smoking because of imitating others and others also tried to persuade me to smoke.” (Village leader, Junan county, former smoker)	Persuasion by and imitation of others	Influence of family and friends	
“I have been smoking since I was 20, influenced by the older generation in my family, many of them smoked.” (Village resident, Pingyin county, current smoker)	Influenced by family members		
“I often played with older kids. They would give me one cigarette when they smoked and gradually I learned to smoke myself.” (Village resident, Liangshan county, current smoker)	Influenced by older kids		
“When I was 41 years old, I had nothing to do and began to learn to smoke. I smoked for fun. I know people hold it against me and I only smoked at home privately. ” (Village resident, Junan county, female current smoker)	Smoke to combat loneliness	Boredom and killing time	
“I don’t have to work every day. Rural villagers have a lot of spare time. Usually people will go by the roadside to have a chat and smoke together to kill time.” (Village resident, Pingyin county, former smoker)	Smoke to kill time		
“I smoked due to the needs of my job [used to be the village military commander]. It is needed for communication and improving relationship. The reasons for villagers to smoke are needs of their jobs and communication custom. It is embarrassing not to offer cigarettes or not accepting cigarettes when offered by others. In order to conduct any business, cigarette gifting has become a custom in the village.” (Village leader, Pingyin county, former smoker)	Communication and Conducting business	Social needs	
“For weddings and funerals, it’s impossible to have no cigarettes. The higher the price and the more quantities of cigarettes provided, the more admired by others.” (Village resident, Junan county, current smoker)	Symbol of politeness and status		
“My husband smokes occasionally. I often remind him to bring cigarettes to give to others when he goes out. I think this helps establish better relationship with others.” (Female village resident, Pingyin county, non-smoker)	Relationship building		

### Ethics

The study was approved by the Research Ethics Board at the Shandong University. Prior to their interviews or discussion sessions, verbal consent to participate was obtained from all participants. All were assured of confidentiality of their information discussed during the interview. Each participant received a high quality towel as a token of appreciation for their efforts.

## Results

In total, 59 subjects participated in the study, including 37 village residents, 12 village doctors, and 10 village leaders (including one who was also a village doctor) (Table 2). By design, nearly all (89%) village residents participated were either former or current smokers. Fifty-eight percent of the village doctors and 80% of the village leaders interviewed also smoked, half of them were current smokers (Table 2).

**Table 2:** **Study participants**

	Village resident (n = 37)		Village leader (n = 10)		Village doctor (n = 12)		Total (n = 59)	
**Age,** mean (range)	43.7 (16–63)*		46.0 (31–64)		46.8 (32–67)			
**Gender**								
Male	35	95%	10	100%	10	83%	55	93%
Female	2	5%	0	0%	2	17%	4	7%
**Years of education**								
0-6	12	32%	0	0%	0	0	12	20%
7-9	22	60%	3	30%	1	8%	26	44%
10-12	3	8%	7	70%	11	92%	21	36%
**Primary occupation**								
Agriculture	18	49%						
Factory worker	13	35%						
Business	6	16%						
**Smoking status**								
Current smoker	22	59%	5	50%	6	50%	33	56%
Former smoker	11	30%	3	30%	1	8%	15	25%
Nonsmoker	4	11%	2	20%	5	42%	11	19%
**Interview type**								
Individual	10	27%	10	100%	12	100%	32	54%
Group	27	73%	0	0%	0	0%	27	46%

*Mean age was calculated based on 9 village residents interviewed individually. One village resident interviewed individually had missing age information. For confidentiality, age information was not asked of focused group participants.

### Why do villagers start to smoke?

Smoking among villagers were reported to vary by age and most prevalent among those aged 40–60 years. Smokers were mostly males. Influenced by traditional culture, very few females smoked and if they did, they usually smoked in private. The most commonly reported reasons for smoking initiation were curiosity, influence of family members or peers, boredom and killing time, and social needs.

#### Curiosity

Curiosity was one of the main reasons for young people to start smoking. When seeing people around them smoking or watching people smoking on television, they wanted to imitate and often thought the appearance of smoking a cigarette is very cool and charming.

*“…Most of smokers who are beginners are adolescents, who started to smoke mainly out of curiosity and were attracted by the ‘cool’ appearance of smoking a cigarette.” (*Village leader, Pingyin, current smoker) *“I don’t enjoy the smell of cigarettes. But I found that the ‘smoking pose’ with two fingers gently gripping the cigarette, inhaling and blowing out smoke rings were very attractive and charming when watching smokers doing that on TV.”* (Village resident, Liangshan, current smoker)

#### Influence of family and friends

Many reported being offered first cigarette during gathering with friends or family, which started them on smoking. Some reported imitation of others, usually someone older than them.

*“I have been smoking since I was 20, influenced by the older generation in my family, many of them smoked*.” (Village resident, Pingyin county, current smoker) “*I often played with older kids. They would give me one cigarette when they smoked and gradually I learned to smoke myself*.” (Village resident, Liangshan county, current smoker)

#### Boredom and killing time

Boredom was another commonly reported reason for smoking initiation. Villagers often have a lot of spare time and they gather around smoking to kill time and socialize.

*“I don’t have to work every day. Rural villagers have a lot of spare time. Usually people will go by the roadside to have a chat and smoke together to kill time.”* (Village resident, Pingyin county, former smoker) For widowed elderly, smoking provides a way to combat the loneliness. *“When I was 41 years old, I had nothing to do and began to learn to smoke.”* (Village resident, Junan county, female current smoker)

#### Social needs

An important reason for smoking was the social needs. In China, it is customary to offer cigarette to others as a gesture of friendship and respect. This practice, referred to as “cigarette gifting” [12, 13], is even more prevalent in rural areas and is regarded as a “must” for conducting any business [12]. Not only not offering cigarettes is considered impolite, refusing cigarettes when offered is also disregarded.

*“I smoked due to the needs of my job [used to be the village military commander]. It is needed for communication and improving relationship. The reasons for villagers to smoke are needs of their jobs and communication custom. It is embarrassing not to offer cigarettes or not accepting cigarettes when offered by others. In order to conduct any business, cigarette gifting has become a custom in the village.”* (Village leader, Pingyin county, former smoker)

### Why do villagers continue to smoke?

Boredom and social needs were not only the reasons for smoking initiation, but also the causes for someone to continue to smoke. In addition, the following three factors were also reasons for villagers to continue to smoke.

#### Limited knowledge about the harm of smoking

Rural villagers had limited knowledge on health hazards associated with active and second-hand smoking. Most reported learning about the health hazards of smoking on the television. Although most villagers were aware that smoking is harmful to one’s health, few specified the harms or diseases caused by smoking when asked. The most frequently mentioned smoking-related health hazard was harm to trachea and some also mentioned harm to the stomach.

*“I know smoking is harmful but don’t know what specific hazards it causes*.” (Village resident, Junan county, current smoker) One village leader reported a misperception by some villagers that “*tracheitis [among smokers] is not a direct effect of smoking but a result of poor quality of the trachea.”* (Village leader, Pingyin county, current smoker)

There was very little agreement on whether second-hand smoking is harmful, and if harmful, whether it is more harmful than active-smoking. Villagers were also unclear about the specific harm caused by second-hand smoking. One described the harm of smoking to others as “*fumigation*”. Some believed smoking inside is harmless to others if “*the room is large*”. Others specifically mentioned the harms to high-risk populations such as pregnant women and children.

There was also a misperception that if one dose not “feel” the harmful effects from smoking, it is not harmful.

*“…The third reason [for me to continue smoking] is that I am fairly healthy and don’t feel much harm of smoking [has hypertension, but experiences no symptoms, and doesn’t care]”* (Village resident, Pingyin county, current smoker)

#### Physical and psychological effect of smoking

Physical and psychological effects derived from smoking, including stress release, relaxation, and anti-depression effect, were also reasons for smoking continuation.

*“Smoking is a kind of relaxation or an excuse. It is reasonable for employees to tell their boss that they want to take a cigarette break. Likewise, one may take a cigarette break when they are tired of working in the field.”* (Village resident, Liangshan county, current smoker) *“When you feel tired while working, smoking can give you a relief to some degree*” (Village resident, Liangshan county, current smoker) *“I began to smoke because of the stress caused by house building work*…” (Village leader, Liangshan county, current smoker) *“I smoke mainly when I feel sad or bored.”* (Village resident, Liangshan county, current smoker)

Others continued to smoke because of their dependence on smoking.

*“I smoke while working and smoke sometimes when I have an urge to smoke while watching TV.”* (Village leader, Liangshan county, current smoker) *“I believe most people smoke not because of addiction to tobacco, but because of psychological dependence on tobacco. If one does not smoke, one feels as if one has lost something or feels annoyed.”* (Village resident, Liangshan county, current smoker)

#### Financial consideration: affordable

Prices of cigarettes or tobacco products were still relatively low and affordable to most villagers. Thus, quitting due to financial reasons were rare, but one successful case was reported. *“In our village there is a man in his 50s who has successfully quit smoking because of the financial burden to support two children in school. But such cases are rare and very few quit smoking because of financial reasons. ”* (Village doctor, Pingyin county, non-smoker)

### What makes villagers want to quit?

Two main reasons that made villagers want to quit: health issues experienced directly or indirectly, and discouragement or dissuasion of smoking from family members or friends

#### Health issues experienced directly or indirectly

Smoking cessation was motivated mainly by health issues experienced either directly or indirectly. Nearly all participants who have attempted or successfully quit smoking reported experiencing some health issues prior to quitting. But those who successfully quit frequently reported significant health events that prompted a visit to a doctor and at doctors’ advice they quit.

*“One of my uncle smoked tobacco. Because of a tongue infection, he accepted the doctor’s suggestion and hasn’t smoked since.”* (Village resident, Liangshan county, former smoker) “*One person had tried to stop smoking for many times but failed. Last year he didn’t feel well. His leg ached and he felt discomfort all over his body. Then he went to the hospital for an examination. The doctor advised him to stop smoking. Otherwise it would hold back the cure of the disease. He then stopped smoking.”* (Village doctor, Liangshan county, non-smoker) *“A man age 54 with a history of smoking for 32 years was found to have lung cancer by CT scan. Just as the doctor was talking with him about his disease, he decided to quit smoking. Afterwards, he never smoked again.”* (Village doctor, Junan county, smoker)

Sometimes, witnessing the dramatic harmful effects of smoking experienced by other smokers can motivate one to quit. *“The direct reason for me to quit smoking was that the former village military commander, who was diagnosed with lung cancer in 1986 or 1987 due to smoking. When he was operated on for his lung cancer, tobacco tar flowed out. I learned a great lesson from his experience.”* (Village leader, Pingyin county, former smoker)

Fire hazard caused by smoking was also reported as a reason. *“I am thinking of quitting because cigarettes often burn my clothes.”* (Village resident, Pingyin county, female current smoker)

#### Discouragement or dissuasion of smoking from family members or friends

Many reported discouragement and dissuasion of smoking from family members or friends as a reason for making quit attempts. Although some short-term effect on smoking cessation were reported, few reported successful quitting long-term because of persuasion by others alone.

*“I don’t remember how many times I attempted to quit. My wife and children all tried to persuade me not to smoke. Each time they asked me, I would stop for as long as 3 days. But afterwards, I would start smoking again.”* (Village doctor, Junan county, smoker)

### Why could not villagers successfully quit?

Almost all current smokers reported desire to quit and many reported multiple quit attempts. Lack of willpower to endure the withdraw symptoms from quitting and to resist the temptation of physical benefits from smoking were believed to be the main reasons for unsuccessful quitting efforts. However, major barriers to smoking cessation were the social acceptance and pressure to smoke among men and the custom of cigarette gifting. In addition, limited resources available to help villagers quit were another important reason.

#### Social environment

A common theme reported by several smokers who had failed attempts was the offering of cigarettes by others after abstinence for a short period time, which caused them to smoke again. Additionally, being surrounded by family members, friends, or coworkers who smoke made it harder to quit.

*“[I] quit one time, but because [I] do not drink, others often jokingly ask [me] ‘is a man who neither smoke nor drink a real man?’ Secondly, because of the house building work, the home owner prepared cigarettes [for us] and [I] just smoked since the cigarettes were free.”* (Village resident, Pingyin county, current smoker)

#### Limited resources to help villagers quit

Lack of knowledge and access to effective and affordable smoking cessation medications of both village residents and village doctors was another barrier. Smoking cessation medications were generally not available in the village clinics. Only one village doctor reported having smoking cessation medications in the clinic and had limited success with them. “*The effect of these medications is still yet to be determined*” (Village doctor, Liangshan county, non-smoker). Another village doctor reported prescribing traditional Chinese medicine for smoking cessation but achieved little success. *“I prescribed some traditional Chinese medicines before and had 2–3 patients tried them. It appeared to have had no effect. This was because they had no intention to quit themselves. Traditional medicine would have had some effect. But they could not make up their mind to quit.”* (Village doctor, Liangshan county, non-smoker)

No participants reported use of any evidence-based western smoking cessation medications. Most quit cold turkey and used candy, nuts, or seeds to fight off carving. Only one reported purchase of some Chinese smoking cessation medication. This medication has unknown efficacy and was expensive, which discouraged its use. Some believed using medication is the only hope for them to quit, but were unaware of any effective medications.

*“I want to quit and feel I had enough of smoking. Last year, I felt smoking was not interesting, so I attempted to quit. I bought “Jie Yan Ling” [a Chinese smoking cessation medication] from a pharmacy in Daguan Village. It cost 10 yuan per package and each package contained 16 [medicine] sticks. I smoked 3–4 sticks a day and used 1–2 packages. There were no discomfort. I did not buy more mainly because the medication was too expensive. However, I still crave smoking when seeing others smoke, so I could not quit.”* (Village resident, Junan county, current smoker) *“I want to quit, but feel that I can’t. If there is smoking cessation medication, I will be able to quit. Otherwise, it will be very difficult to quit at my age without the help of a medication*.” (Village resident, Liangshan County, current smoker)

There was also misperception that all smoking cessation medications are not effective.

*“Taking smoking cessation medications does not help. It has been broadcasted through the loud speaker that these medications are not effective.”* (Village leader, Junan county, current smoker)

No specialized smoking cessation counseling services were available at the village clinics. Interviews of village doctors found that most asked patients’ smoking history and/or giving advices to quit or smoke less only when the patients came to the clinics smoking or had respiratory-related illnesses. Most doctors expressed pessimism about their advice or advice by others in general and believed willingness to quit, strong willpower, degree of addition, and patients’ health status are the determining factors.

*“The key to smoking cessation in villages is individual’s physical and mental health status. It depends on a person’s willingness to quit and willpower. It is unnecessary to train health care providers [on smoking cessation measures]. The strategies should be personalized.”* (Village doctor, Pingyin county, former smoker) *“When villagers come to the clinic, some were asked about their smoking status, some were not asked. Sometime if it is tracheitis, [I] would ask. There is no difference between rural or urban areas. There are no good approaches [to control smoking] in rural areas. Many know it is harmful, but they still smoke. Propaganda does not work. Might work among older people. Young people won’t listen*.” (Village doctor, Liangshan county, non-smoker)

### Barriers to implementation of tobacco control policies

Tobacco control activities at villages were rare and infrequent. Commonly reported activities were smoking cessation-related posters and blackboard bulletins at village gathering places or in village clinics. Pessimism regarding the effectiveness of these activities were widely shared by participants.

When asked about implementing population-wide tobacco control policies such as tobacco-free villages or establishment of monetary punishment for smoking in villages, most doubted their practicality. The perceived barriers included: 1) large base of smokers; 2) village residents diversely located with few “public places” and decentralized locations for smoking (e.g. in the field, at home), making it hard to monitor and control; 3) difficulty monitoring young people who work outside of village; 4) social norm for cigarette gifting; 5) lack of ties to performance. Some argued for stepwise process instead of drastic measures like tobacco-free villages and suggested starting with smaller units such as tobacco-free households. Reluctance to interfere was also clear from the interviews and FGDs because most regarded smoking as a personal affair, a habit that may cause harm to the smokers themselves without affecting others. Such an attitude can be clearly seen in one interviewee’s statement,

*“I am annoyed when seeing others smoking, but will not interfere. They may become angry if I do. Smoking is one’s habit. Others can’t interfere.”* (Village doctor, Junan county, non-smoker)

Suggestions for effective tobacco control included 1) more smoking hazards education with graphical demonstrations such as diseased lungs by medical professionals or even X-rays of smokers’ own lungs to allow the smokers see the damage to their own body from smoking, 2) provide effective smoking cessation medications or electronic cigarettes, and 3) targeted education of wives and children to persuade husbands or parents not to smoke. Some also suggested significant increases in cigarette price, although others doubted its effectiveness because “*those who want to smoke will find a way*” (Village resident, Liangshan county, former smoker). Some believed only drastic measures such as closing tobacco factories will be effective.

## Discussion

Results from this study of rural villagers in an eastern province of China suggested that tobacco control efforts in rural villages should focus on the following areas:

1. Combating cigarette gifting custom. In nearly all cases, the first cigarette was offered by a family member, a friend, or a coworker during social gatherings or business functions, which started them on smoking. These findings reflected the unique cigarette sharing and gifting practice in China, in which, as a gesture of friendship and respect, an individual cigarette is offered to others for immediate consumption during social and business functions or cartons of cigarettes are given as gifts during holidays [13]. Not only offering cigarettes is considered a proper etiquette during business and social functions, accepting cigarettes when offered is also expected and refusing such offer is often regarded as being disrespectful [13]. As a result, cigarette gifting is reported to be a major barrier to smoking cessation in this study, by making cigarettes easily assessable (without purchasing cigarettes personally) and imposing pressure to give and accept cigarettes and to smoke to “fit in”. These findings are consistent with many other studies [12, 13, 23]. As argued by other researchers [13, 23], changing this culture of cigarette gifting is essential to smoking cessation in China, particularly in rural areas where such practice is more prevalent [13]. While mass media campaigns have reduced cigarette gifting practice in some large cities, such campaigns are not yet extended to rural China [13]. This study and others [12, 13]) found that cigarette gifting custom is still deeply rooted and widely accepted in rural villages, making it difficult to achieve large scale smoking cessation among rural village residents.
2. Routine smoking screening and counseling. The most important reason for quitting successfully was a detrimental health problem. Doctors’ advice played an important role in these successes as almost all who quit successfully reported quitting at doctors’ advice after they experienced a health problem. However, interviews with village doctors found that not all village doctors routinely provide smoking screening or cessation advice to patients who are smokers. Some only asked about history of smoking if a patient had respiratory illnesses or provided cessation advice to patients if they had respiratory illnesses or were smoking when came to the clinic, suggesting missed opportunities for village doctors to provide smoking screening of all patients and cessation interventions to all smokers. This finding suggests that providing routine health screening and smoking cessation counseling to rural village residents by healthcare providers are important to prevention and early detection of smoking-related diseases and reduction of smoking-related morbidity and mortality. As one participant suggested, seeing the damage from smoking to a smoker’s own lung using a chest x-ray may motivate him/her to quit.
3. Smoking cessation among health care providers. Although not designed to be a representative sample, over half (58%) of the village doctors interviewed were current or former smokers. The high prevalence of smoking among village doctors observed in our sample is consistent with previous studies that have found high smoking prevalence among Chinese physicians (14%-64%) [24] and physicians in other developing countries [25], compared to physicians in developed countries. Being a current smoker is associated with significantly reduced probability of offering smoking cessation advice to patients by physicians [26], which may explain the overall pessimistic view among the village doctors regarding the effectiveness of their advice and their reluctance to offer such advice to all patients. Our study contributes to the literature by providing insights on rural village doctors, whom have not been studied previously [24]. Unlike doctors in the province, city, or county hospitals, village doctors received much less formal education [27]. This and combined with the overall limited training in tobacco control measures among health care providers in China [24], may further hinders’ village doctors’ ability to implement effective tobacco control measures in villages.
4. Targeted education of family members particularly wives and children to help their husbands and fathers quit. Previous studies have suggested that encouragement by family members is a significant motivator for smoking cessation [28]. However, this study found that although many attempted to quit at the advice of their family members, relapses were common and few were able to quit completely and for long-term unless accompanied by significant health issues. Other studies of rural village residents also found that even knowing smoking is unhealthy, family members may not discourage and sometimes even encourage other family members to smoke to maintain both intra-family (avoid conflicts between family members) and extra-family relations (for business and social networking) [12, 29]. As smoking is primarily a male behavior in China, this lack of effectiveness of cessation advices provided by family members (mostly women) may have reflected the traditional male-dominance gender norm in rural China, which may prevent women from effectively challenging men’s smoking [22]. Even among pregnant women, studies have found that while many may limit home smoking to protect their unborn children, few ask smoking family members to quit or to reduce cigarette consumption [11, 30, 31]. On the other hand, parent-smoker role conflict and concerns of children’s health are an important motivation for smoking parents to quit [32, 33], suggesting that interventions targeting children to persuade parents to quit or providing smoking cessation counseling to parents during children’s healthcare visits [29] may be more effective strategies to smoking cessation among parents.
5. Effectively regulate local tobacco products. Another barrier to smoking cessation in China is the high affordability and wide price range of cigarettes. An analysis of the 2008–2011 Global Adult Tobacco Survey of 15 countries found China ranked the 3rd in both affordability and wideness of cigarette price range [34]. This study found that rural villagers also consider cigarettes affordable and rarely quit because of financial reasons unless there are significant competing financial needs. This indicates that increasing cigarette price has the potential to curb cigarette consumption in this less affluent population. However, as this study revealed, availability of low-cost alternatives, particularly local tobacco products in rural areas, makes it easy to switch to cheaper alternatives without significant reduction in quantity consumed and thus reduces the potential effect of price/tax hike on cigarette consumption. Moreover, other studies of rural village residents found that because of the re-gifting custom, expensive cigarettes are traded as currencies and can be exchanged for (or for money to purchase) cheaper alternatives [12]. Smoking expensive cigarettes is also regarded as a symbol of affluence as reflected in one of the participant’s boasting of his experience with an expensive brand of cigarette. For these reasons, moderate cigarette price increases are not likely to achieve significant success in rural villages unless cigarette gifting custom could be reduced and cheap local products can be effectively regulated.
6. Educating both village residents and doctors on effective smoking cessation medications as well as increasing access to these medications. This study found that both smokers and doctors in Chinese rural villages are lacking knowledge of effective smoking cessation medications. The most common smoking cessation method reported is quitting cold turkey and using melon seeds or candies to fight off cravings. This method requires significant self-discipline and determination. First-line smoking cessation agents such as nicotine replacement therapy, bupropion, and varenicline are recommended to maximize smoking cessation [35]. However, use of these agents is low in China [36], despite their approval for use in Asian countries since 2007 and their demonstrated efficacy in clinical trials using Chinese participants [37, 38]. In this study of Chinese rural villagers, we found even lower use of smoking cessation agents, with only one rural village clinic reported availability and prescription of some unknown smoking cessation medications, one other doctor reported prescribing traditional Chinese medicine, and one village resident reported use of some over-the-counter Chinese smoking cessation medication, none of which, however, achieved success in long-term abstinence. More educations of effective smoking cessation treatment to both village residents and healthcare providers are warranted.

### Limitations

Participants were rural villagers in an eastern province of China and therefore findings are representative of rural villagers in this area and may not generalize to all rural residents in China. To protect confidentiality, minimum demographic characteristics were collected which prevented further subgroup analysis. In some counties, we were only able to conduct one FGD. Therefore, more diverse demographics may have been included to solicit more diverse views. However, when it became obvious that group dynamics in several discussion groups inhibited full participation by some discussants, we conducted semi-structured face-to-face interviews with additional rural residents to allow participants to speak freely about their experiences in a more confidential manner and reached information saturation and redundancy after that. We relied on village leaders and doctors’ recommendations to identify potential study participants because we believed that the village leaders/doctors are most familiar with the local culture and residents. While this approach may have increased the efficiency of identifying participants who are most familiar with smoking behavior of their fellow villagers and are willing to participate, it is possible views represented in this study may be biased if their choices/recommendations were biased.

## Conclusions

This study highlighted the need to develop tobacco control measures that reflect the unique culture in rural China. Smoking cessation measures are not likely to achieve large scale effect unless the prevailing cigarette sharing and gifting custom is drastically changed. In addition, there is a lack of true understanding of the harm of smoking and second-hand smoking among the villagers and a lack of access to and knowledge of effective smoking cessation tools among both smokers and village doctors, suggesting that more educations of both village residents and healthcare providers are needed.

In 2005, China ratified the WHO Framework Convention on Tobacco Control (FCTC), which embraces the proven tobacco control measures such as higher taxes, strong warning labels, smoke-free air laws, aggressive public education campaigns, packaging restrictions and restrictions on tobacco industry marketing [39]. However, implementation of these measures nationwide in China has been difficult with little effect on the overall tobacco consumption [40, 41]. Many barriers were identified [41], with the most important being the strong resistance from the tobacco industry and lack of sufficient government support. [40–42] In addition to these system-level barriers, rural villages in China facing additional challenges to effectively implement these evidence-based tobacco control measures. For instance, smoke-free air laws are hard to implement, monitor and enforce in rural villages because there are fewer clearly defined public areas. In addition, price of cigarettes is still very low in the rural areas; to achieve significant reduction in smoking, substantial increases in price/tax may be needed. Moreover, cheaper local tobacco products would need to be effectively regulated to prevent smokers from switching to or substituting these cheaper alternatives to achieve real reduction in consumption of tobacco products. On the other hand, our study suggests that pictorial warning on the packaging or aggressive education program that include visual illustration of physical damage from smoking to a human body could be particularly effective tools to encourage rural village smokers to quit.
